# A Case of Allergic Bronchopulmonary Mycosis Caused by Cordyceps farinosa, a Species of Caterpillar Fungi

**DOI:** 10.7759/cureus.68366

**Published:** 2024-09-01

**Authors:** Yuta Sakano, Taro Okumura, Tomomi Kofuku, Saki Kidaka, Yugo Nakata, Satoshi Katsura, Kenichi Goto, Michio Shigematsu

**Affiliations:** 1 Department of Pulmonology, Sumitomo Hospital, Osaka, JPN; 2 Department of Clinical Laboratory Technology, Sumitomo Hospital, Osaka, JPN

**Keywords:** dna sequencing, allergic diseases, entomopathogenic fungi, cordyceps farinosa, allergic bronchopulmonary mycosis

## Abstract

A female patient in her thirties presented with persistent cough and sputum, unresponsive to conventional treatments. Chest imaging showed infiltrative opacities and high attenuation mucus. On laboratory examination, eosinophil counts and immunoglobulin E were elevated. *Cordyceps farinosa*,a species of caterpillar fungi, was identified by bronchoscopy and subsequent DNA sequencing from the mucus plug. The symptoms improved after the removal of mucus plugs and cessation of exposure to the work environment. Allergic bronchopulmonary mycosis (ABPM) caused by *C. farinosa* has not been reported, and its pathogenicity is not well recognized. Herein, we report this case to understand the disease spectrum of ABPM and the pathogenicity of this rare fungi.

## Introduction

Allergic bronchopulmonary mycosis (ABPM) is a hypersensitivity-mediated disease caused by environmental fungi colonization of the lower respiratory tract, characterized by bronchitis, eosinophilia, bronchiectasis, and mucus plugs [1]. ABPM due to *Aspergillus* sp. (allergic bronchopulmonary aspergillosis (ABPA)) is the most common type [2]. Cases of ABPM caused by non-*aspergillus* fungi such as *Schizopyllum commune* (*S. commune*) have been increasing [2]. However, there are few reports of causative fungi of non-*aspergillus* ABPM other than *S. commune*, and the clinical course is unclear. Therefore, some ABPM cases are difficult to diagnose definitively because the causative fungus cannot be identified.

In this study, we experienced a case of ABPM caused by *Cordyceps farinosa* (*C. farinosa*), a species of caterpillar fungi. At the time of the visit, there were no established diagnostic criteria for ABPM. However, the first diagnostic criteria for ABPM were reported in 2021 [1], so we could make a definitive diagnosis. To our knowledge, no case of ABPM caused by *C. farinosa* has been reported; therefore, this case could help in understanding the disease spectrum of ABPM.

## Case presentation

A female, non-smoker patient in her thirties visited our hospital in November 2012. The patient presented with cough and sputum, persisting since August 2012. No fever, nasal discharge, headache, joint pain, eruption, or abnormalities in breath sounds were noted. The patient was treated with a salmeterol inhaler (100 µg), fluticasone propionate inhaler (500 µg), and montelukast (10 mg); however, her symptoms did not improve. The patient had atopic dermatitis and no allergies to medicine or food. The patient’s family had no allergic diseases. The patient resided in a two-year-old reinforced concrete condominium and was free from dampness and mold. There were no pets and no recent international travel or outdoor experience. The patient had been engaged in breeding mice and rats in a chemical laboratory in the mountains for a year prior to the visit. The mice were fed commercial pellets, and no insects were used. The mice were in cages lined with paper chips. When the patient entered the breeding room, her symptoms worsened repeatedly.

**Figure 1:**
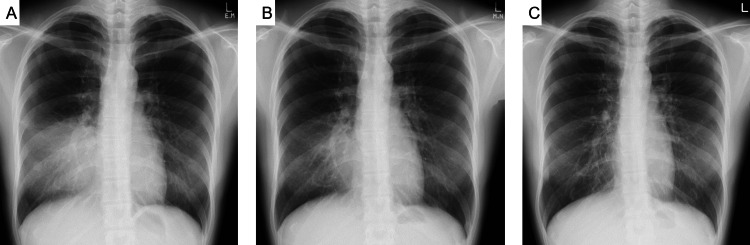
Chest X-ray images during follow-up. A. Chest X-ray on admission showed consolidation in the right lower lung field. B. Eleven days after bronchoscopy, the consolidation showed improvement. C. The shadow had disappeared 53 days after bronchoscopy.

**Figure 2:**
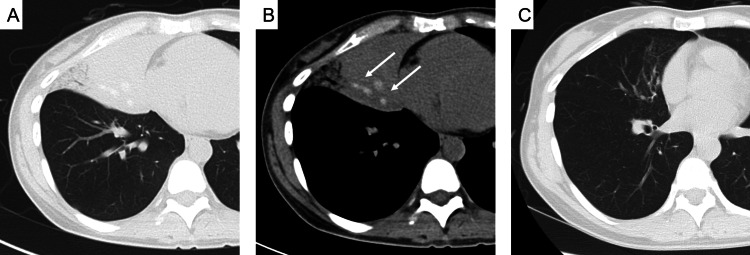
Chest computed tomography during follow-up. A. Chest computed tomography showed infiltrative opacities in the left S^4-5^. B. In the mediastinal window, the left B^4-5^ bronchus are dilated and filled with high attenuation mucus (arrows). C. Six months later, the mucus plug and infiltrative shadow had disappeared, and only bronchiectasis remained.

On admission, chest X-ray showed consolidation in the right lower lung field (Fig. 1A). The patient was treated with levofloxacin (500 mg/day for a week), suspecting bacterial pneumonia; however, the lung shadows did not improve. In addition, the patient developed chest pain when coughing or breathing in. Chest computed tomography (CT) showed infiltrative opacities in the left S^4-5^ (Fig. 2A) and high attenuation mucus (HAM) in the left B^4-5^ bronchus, which were dilated in the mediastinal window (Fig. 2B). On laboratory examination, eosinophil counts, immunoglobulin E (IgE) and C-reactive protein (CRP) were elevated (Table 1). Radioallergosorbent tests for specific IgE antibodies for *Aspergillus*, mites, pollen, and rat hair were positive. *Aspergillus* galactomannan antigen and *Aspergillus* immunoglobulin G (IgG) antibody are negative. Pulmonary function test showed no impairment of respiratory function.

**Table 1:** Laboratory findings on admission. ALP, alkaline phosphatase; ALT, alanine aminotransferase; AST, aspartate aminotransferase; BUN, blood urea nitrogen; CRP, C-reactive protein; IgE, immunoglobulin E; LDH, lactate dehydrogenase; RBC, red blood cell; WBC, white blood cell

Lab	Result	Reference range
Hematological data		
WBC	9,400*	(3,300-8,600) /µL
Neutrophils	73.0	(42.4-75.0) %
Eosinophils	11.6*	(0.4-8.6) %
Basophils	0.5	(0.2-1.4) %
Monocytes	5.3	(3.3-9.0) %
Lymphocytes	9.6*	(18.2-47.7) %
RBC	370×10^4^*	(380-500×10^4^) /µL
Hemoglobin	11.3*	(11.5-15.0) g/dL
Hematocrit	33.0*	(35-45) %
Platelets	27×10^4^	(15-35×10^4^) /µL
Serological data		
Total IgE	3,860*	(<173) IU/mL
Biochemical data		
AST	12*	(13-33) IU/L
ALT	8	(6-30) IU/L
LDH	183	(119-229) IU/L
ALP	224	(115-359) IU/L
BUN	10	(8-20) mg/dL
Creatinine	0.53	(0.36-1.06) mg/dL
Na	139	(135-147) mEq/L
K	4.4	(3.6-5.0) mEq/L
Cl	101	(98-108) mEq/L
CRP	4.9*	(<0.3) mg/dL

Two weeks after admission, the patient underwent bronchoscopy. The right middle lobar bronchus was obstructed by a brown mucus plug. The mucus plug was removed and analyzed for bacterial and fungal cultures. After the removal of the mucus plug, airway secretions could be aspirated, and the right B^4-5^ opening was confirmed. Eleven days after bronchoscopy, cough and sputum improved. A chest X-ray taken the same day showed improvement of the infiltrative shadow in the right lower lung field (Fig. 1B). Chest X-ray 53 days later showed the disappearance of the infiltrative shadow (Fig. 1C). In parallel with the changes in the chest X-ray, laboratory examination also showed improvement in eosinophil counts and CRP. IgE levels decreased but were still higher than the reference range (Table 2). Later, the patient retired from her mouse breeding job. Six months later, chest CT showed that the mucus plugs and infiltrative shadow had disappeared, and only bronchiectasis in the middle lobe remained (Fig. 2C). Her symptoms had resolved, and she ended her outpatient visits. No recurrence has since been observed.

**Figure 3:**
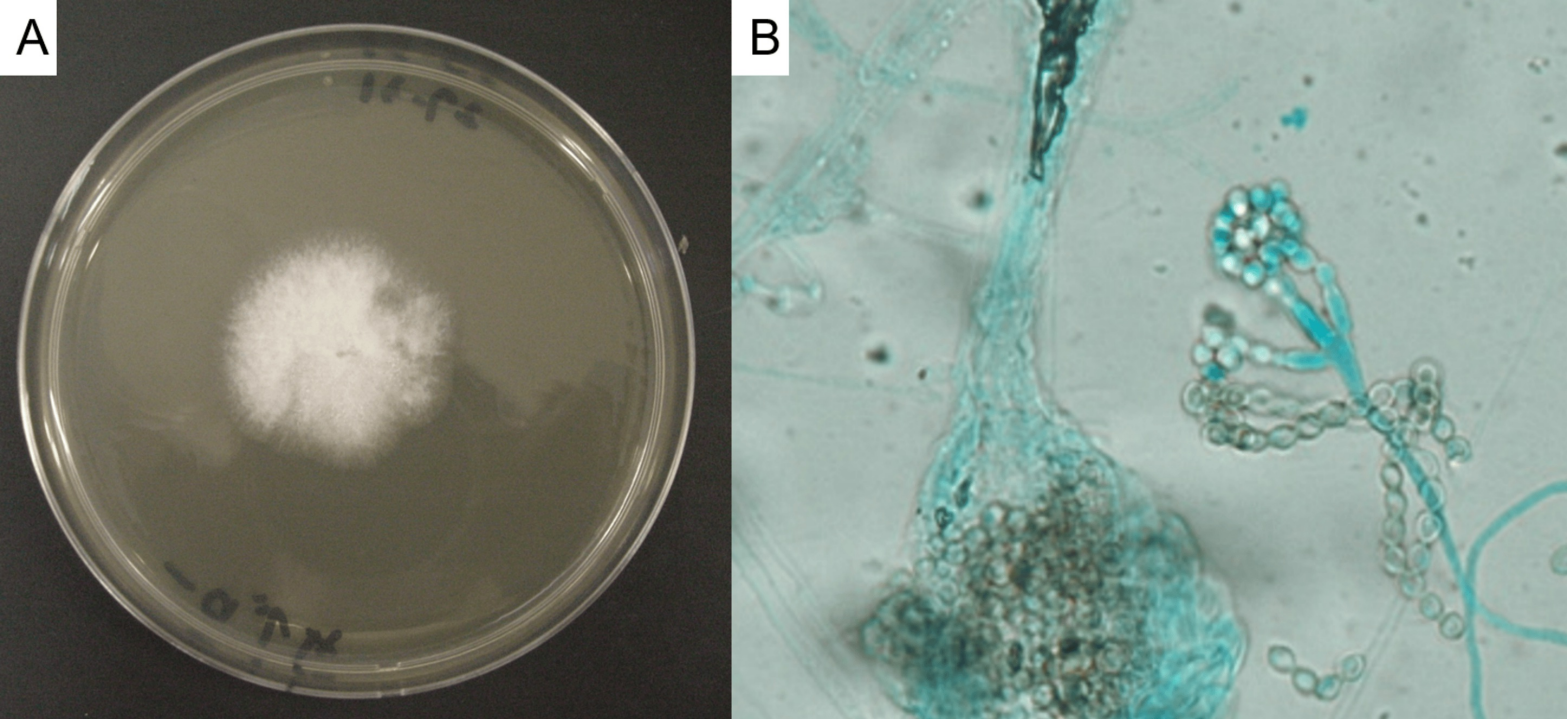
Macroscopic and microscopic appearance of the fungs. A. Macroscopic appearance of the culture of lower airway samples. On day 3 of culture, a white fungus with a fluffy-like appearance rapidly grew on sabouraud dextrose agar medium at a temperature of 25°C. B. On scotch tape preparation, filamentous fungus was observed, but morphological identification was difficult.

Lower airway samples obtained by bronchoscopy were continuously cultured aerobically on a Sabouraud dextrose agar medium at a temperature of 25°C. On day 3 of culture, white colonies with a fluffy-like appearance developed on the medium (Fig. 3A). Filamentous fungus was observed on scotch tape preparation; however, morphological identification was difficult (Fig. 3B). Deoxyribonucleic acid (DNA) sequencing of internal transcribed spacer (ITS) region was performed in Kobe City Medical Center General Hospital. The fungus was identified as *C. farinosa*. The sample was analyzed by extracting DNA using MORA-EXTRACT (Kyokuto Pharmaceutical Industrial Co., Ltd., Tokyo, Japan). The 5.8 S rDNA and the flanking ITS regions (ITS1 and ITS2) were amplified using the primers ITS5 and ITS4 using a polymerase chain reaction (PCR) thermal cycler system Thermal Cycler (LifePro, Bioer LifeScience Japan Co., Ltd., Kobe, Japan). The sequencing was performed by an Applied Biosystems ® 3500 genetic analyzer (Thermo Fisher Scientific Inc, TX, US). On day 24 of the culture, *Mycobacterium avium* was detected. The patient did not receive specific treatment for *Mycobacterium avium* complex (MAC) lung disease because no respiratory symptoms and imaging changes appeared during follow-up. When the fungus was identified, there were no established diagnostic criteria for ABPM. The diagnostic criteria of Rosenberg [3] and the International Society for Human and Animal Mycology (ISHAM) [4] were created for ABPA; therefore, a definitive diagnosis could not be made. In 2021, the diagnostic criteria for ABPM (Japanese Society of Allergology) were reported [1]. Since more than six items were positive according to the requirements (Table 3), a definitive diagnosis of ABPM caused by *C. farinosa* was made.

**Table 2:** Course of laboratory data related to ABPM. ABPM, allergy bronchopulmonary mycosis; CRP, C-reactive protein; IgE, immunoglobulin E

Lab	On admission	Eleven days after bronchoscopy	53 days after bronchoscopy	Reference range
Eosinophil counts	1,102*	266	288	(70-450) /µL
Total IgE	3,860*	2,880*	2,110*	(<173) IU/mL
CRP	4.9*	0.12	0.26	(<0.3) mg/dL

**Table 3:** Clinical diagnostic criteria for ABPM in patients without cystic fibrosis. ABPM, allergy bronchopulmonary mycosis; CT, computed tomography; IgE, immunoglobulin E; IgG immunoglobulin G. Filamentous fungi in criteria 4 to 6 should be identical. Patients that meet six or more of these criteria are diagnosed with ABPM. The clinical course of the patient met diagnostic criteria #1, #2, #3, #6, #8, #9 and #10. This criteria were reported by Asano et al. in 2021 [2].

Clinical items of the diagnostic criteria for ABPM in patients without cystic fibrosis.	
1.	Current or previous history of asthma or asthmatic symptoms
2.	Peripheral blood eosinophilia (>500 cells/mm^3^)
3.	Elevated total serum IgE levels (>417 IU/mL)
4.	Immediate cutaneous hypersensitivity or specific IgE for filamentous fungi
5.	Presence of precipitins or specific IgG for filamentous fungi
6.	Filamentous fungal growth in sputum cultures or bronchial lavage fluid
7.	Presence of fungal hyphae in bronchial mucus plugs
8.	Central bronchiectasis on CT
9.	Presence of mucus plugs in central bronchi, based on CT/bronchoscopy or mucus plug expectoration history
10.	High attenuation mucus in the bronchi on CT

## Discussion

ABPM is a disease caused by the development of certain fungi, such as *Aspergillus* sp. or *S. commune*, in the bronchi, for which human body temperature is the optimal growth temperature [5]. The development of ABPM requires the inhalation of environmental spores of fungi and their growth in the respiratory tract. Therefore, fungi are required to have their spores smaller than 5 µm and to be able to germinate at human body temperature [6,7]. The clinical symptoms of ABPM include cough, sputum, shortness of breath, and chest pain. Although asthma was once considered an essential complication, it has been reported that approximately 20% of ABPM cases had no asthma [8,9]. Laboratory examination is characterized by increased peripheral eosinophil counts and serum total IgE. In addition, specific IgE and IgG antibodies to filamentous fungi are positive, but the species that can be measured are limited. The most common imaging features include central bronchiectasis and intrabronchial mucus plugs in chest CT, which are called for HAM and known to be the most specific findings in ABPM, especially in women, severe cases, and ABPM caused by *S. commune* [9].

This case is an ABPM caused by *C. farinosa* (formerly *Paecilomyces farinosus*), which has not been reported in the past. Data on ABPM caused by other than *Aspergillus* sp. are limited. Chowdhary et al.’s large-scale literature search reported that *Candida albicans*, *Bipolaris* sp., and *S. commune* accounted for 84% of ABPM [10]. The ABPM study group in Japan reported that *S. commune* accounted for 63%, followed by *Penicillium* sp. and *Mucor* sp. at 13%, indicating wide regional differences in the fungi that cause ABPM [11].

**Figure 4:**
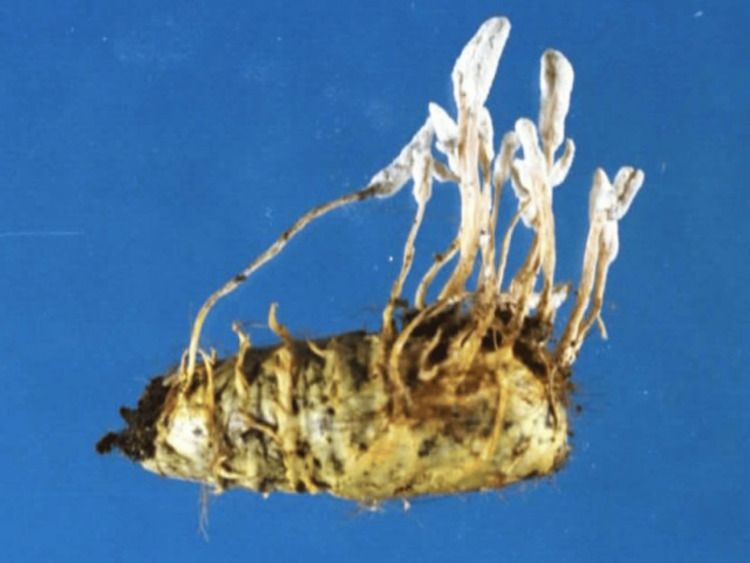
Photograph of lepidopteran pupae infected with Cordyceps farinosa. The host is covered with white or yellow mycelium and often develops a conidiophore.

*C. farinosa* is a parasitic fungus for larvae and pupae of butterflies (Fig. 4), a closely related species of *Cordyceps militaris*, and is used as a traditional herbal medicine in East Asia [12]. It is often referred to as "winter worm, summer grass" because the fungus parasitizes and mummifies insect larvae during the winter and then produces a fruiting body that emerges from the host in the summer. *C. farinosa* is thought to exist in the soil as spores until infection of the host is established; however, the detailed process remains unknown. The mycelium of *C. farinosa* is powdery, white to yellowish white, bearing complex conidial structures and sometimes numerous synnemata. Its vegetative hyphae are septate, hyaline, branched, smooth-walled, and 1.6-4.0 μm wide [12].

There are few reports of *C. farinosa* being pathogenic to humans, and only one case of maxillary sinus mucocele with fungal ball has been reported [13]. For the development of ABPM, inhalation of viable fungi as conidia, not their hyphae fragments, and their germination in the lower airways is essential. Thus, fungal conidia must be small enough to reach the lower respiratory tract [14]. Conidia of *C. farionsa* is 2.4-4.8 × 1.6-3.2 μm and ovoid to cylindrical [12]. The size of this conidia is similar to that of *Aspergillus* sp. (3-6 μm) and *S. commune* (3-4 × 1-1.5 μm). In addition, the temperature range in which *C. farinosa* can develop is from 5°C to 30°C [15]. It is reasonable that *C. farinosa* can colonize in bronchi, considering that *Aspergillus niger* and *S. commune*, well-known causative fungi of ABPM, are able to grow at 30°C and from 30°C to 35°C, respectively. Therefore, this case is the first report showing that *C. farinosa* is pathogenic to humans as ABPM.

In this case, the work environment was assumed to be the cause of the onset of ABPM. The patient lived in an urban area and had no history of living in an environment where she could have been exposed to fungus. Although we were not able to conduct an on-site environmental survey, since the workplace was located in a forested mountainous area and the symptoms were exacerbated in the mouse breeding site, it was assumed that the causative fungus was present in the mouse cage or environment surrounding the workplace. The fact that the patient retired from work after admission and has not had a recurrence of ABPM in the years supports that the disease was caused by that environment. The clinical symptoms were relieved after bronchoscopic removal of the plugs. Although oral steroids or antifungal agents are the standard treatment for ABPM, a case has been reported in which the symptoms were improved only by the removal of the plugs [16]. Moreover, it has been reported that isolation from the causative environment and removal of fungi from the living environment is also effective in allergic diseases, such as asthma or ABPM, which is consistent with the clinical course of this case [17]. In addition to ABPM, *Mycobacterium avium* was co-infected in this patient. non-tuberculous mycobacteria (NTM) is a major complication in patients with ABPA, and steroid therapy for ABPA is a risk for worsening NTM [18]. Therefore, it is fortunate that ABPM improved and MAC disease did not develop in this patient after mucus plug removal and environmental isolation alone.

At the time of the patient's examination, there were no established diagnostic criteria for ABPM. The patient did not have asthma typical of ABPA, and in addition, the causative fungus was not *Aspergillus* sp., making a definitive diagnosis difficult. Rosenberg and ISHAM diagnostic criteria are widely used for ABPA [3,4], but these criteria assume the presence of asthma or cystic fibrosis and elevated serum IgE. By contrast, a survey of patients with ABPM in Japan revealed that some cases lacked typical asthma symptoms or elevated IgE, and it was reported that many cases did not fit the conventional diagnostic criteria [19]. Therefore, in 2021, Asano et al. reported new diagnostic criteria for ABPM that included filamentous fungi other than *Aspergillus* sp. (Table 2) [1]. The sensitivity and specificity of these criteria are 95% and 97%, respectively, superior to those of the Rosenberg and ISHAM diagnostic criteria. Including fungi other than *Aspergillus* sp. in the criteria component for the first time and expanding the criteria for clinical symptoms to asthmatic symptoms, including cough, sputum, and shortness of breath, made the criteria clinically useful. With the release of the new diagnostic criteria, we can officially confirm the definitive diagnosis of ABPM caused by *C. farinosa* in this case. Therefore, this report demonstrates the usefulness of the diagnostic criteria.

## Conclusions

We were able to diagnose ABPM due to *C. farinosa*, which could not be identified morphologically, by performing DNA sequencing on the fungus. Further studies are needed to conclude whether this fungus is indeed a cause of ABPM. Because of the wide variety of fungi that cause ABPM, there are no specific antigens or antibody examinations for each fungus, making it difficult to make a definitive diagnosis. Therefore, the identification of the causative fungus by DNA sequencing should be actively considered, not only to make a definitive diagnosis but also to provide a more accurate understanding of the disease spectrum of ABPM.
